# Circulating sphingolipids and relationship to cardiac remodelling before and following a low-energy diet in asymptomatic Type 2 Diabetes

**DOI:** 10.1186/s12872-023-03623-y

**Published:** 2024-01-03

**Authors:** Emer M. Brady, Thong H. Cao, Alastair J. Moss, Lavanya Athithan, Sarah L. Ayton, Emma Redman, Stavroula Argyridou, Matthew P. M. Graham-Brown, Colleen B. Maxwell, Donald J. L. Jones, Leong Ng, Thomas Yates, Melanie J Davies, Gerry P. McCann, Gaurav S. Gulsin

**Affiliations:** 1 Department of Cardiovascular Sciences, University of Leicester, NIHR Leicester Biomedical Research Centre, Glenfield Hospital, Leicester, LE3 9QP UK; 2 Leicester Van Geest Multi-Omics Facility, University of Leicester, Leicester, UK; 3 Diabetes Research Centre, NIHR Leicester Biomedical Research Centre, Leicester General Hospital, Leicester, UK

**Keywords:** Diabetic cardiomyopathy, Stage B heart failure, Sphingolipids, Ceramide, Cardiac MRI

## Abstract

**Background:**

Heart failure with preserved ejection fraction (HFpEF) is a heterogenous multi-system syndrome with limited efficacious treatment options. The prevalence of Type 2 diabetes (T2D) continues to rise and predisposes patients to HFpEF, and HFpEF remains one of the biggest challenges in cardiovascular medicine today. Novel therapeutic targets are required to meet this important clinical need. Deep phenotyping studies including -OMIC analyses can provide important pathogenic information to aid the identification of such targets. The aims of this study were to determine; 1) the impact of a low-energy diet on plasma sphingolipid/ceramide profiles in people with T2D compared to healthy controls and, 2) if the change in sphingolipid/ceramide profile is associated with reverse cardiovascular remodelling.

**Methods:**

Post-hoc analysis of a randomised controlled trial (NCT02590822) including adults with T2D with no cardiovascular disease who completed a 12-week low-energy (∼810 kcal/day) meal-replacement plan (MRP) and matched healthy controls (HC). Echocardiography, cardiac MRI and a fasting blood for lipidomics were undertaken *pre*/*post*-intervention. Candidate biomarkers were identified from case–control comparison (fold change > 1.5 and statistical significance *p* < 0.05) and their response to the MRP reported. Association between change in biomarkers and change indices of cardiac remodelling were explored.

**Results:**

Twenty-four people with T2D (15 males, age 51.1 ± 5.7 years), and 25 HC (15 male, 48.3 ± 6.6 years) were included. Subjects with T2D had increased left ventricular (LV) mass:volume ratio (0.84 ± 0.13 vs. 0.70 ± 0.08, *p* < 0.001), increased systolic function but impaired diastolic function compared to HC. Twelve long-chain polyunsaturated sphingolipids, including four ceramides, were downregulated in subjects with T2D at baseline. Three sphingomyelin species and all ceramides were inversely associated with LV mass:volume. There was a significant increase in all species and shift towards HC following the MRP, however, none of these changes were associated with reverse cardiac remodelling.

**Conclusion:**

The lack of association between change in sphingolipids/ceramides and reverse cardiac remodelling following the MRP casts doubt on a causative role of sphingolipids/ceramides in the progression of heart failure in T2D.

**Trial registration:**

NCT02590822.

**Supplementary Information:**

The online version contains supplementary material available at 10.1186/s12872-023-03623-y.

## Highlights

• This study sheds light on the emerging link between dysregulated sphingolipid/ceramide metabolism and the development of heart failure (HF) in individuals with type 2 diabetes (T2D).

• Leveraging the gold standard assessment tool, cardiac MRI, this study is one of the first to explore plasma sphingolipid/ceramide in relation to measures of cardiovascular structure and function.

• The findings reveal that working-aged adults with T2D and Stage A/B HF exhibit disrupted fatty acid metabolism, characterized by reduced levels of long-chain polyunsaturated sphingomyelin/ceramide species.

• Importantly, this study challenges the notion of a causative role of sphingolipids/ceramides in the progression of heart failure in T2D.

**Supplementary Information:**

The online version contains supplementary material available at 10.1186/s12872-023-03623-y.

## Background

Type 2 diabetes (T2D) is recognised as one of the most important risk factors for heart failure (HF) [1]. Indeed, the recent universal guidelines for definition of HF has classified all people with T2D as Stage A “at risk” of HF and those with asymptomatic cardiac structural and/or functional alterations as Stage B HF [2]. People with T2D have a propensity for HF with preserved ejection fraction (HFpEF) [3], a heterogeneous clinical syndrome [4], which remains one of the biggest challenges in cardiovascular medicine, given the limited efficacious treatment options. Novel therapeutic targets are required to meet this important clinical need. Deep phenotyping studies including -OMIC analyses can provide information on the underpinning pathogenic mechanisms early in a disease course, track response to intervention and thus aid the identification of such targets [5] .

Dysregulated lipid pathways within cardiomyocytes are considered a potential pathogenic process in the development of HFpEF in the context of T2D [6]. Moreover, complex sphingolipids, and more specifically, dysregulation of ceramide and sphingolipid metabolism are thought to have a cardiotoxic role in the pathogenesis of HF [7–10]. In a small subset of the Alberta Heart study, circulating levels of 14 species of sphingomyelins in people with symptomatic HFpEF (10/24 with T2D) were lower than controls without HF. Further, logistic regression analysis showed that sphingomyelin (C20:2) could discriminate between those with HFpEF and the non-HF controls [11]. Whether or not such signals can be identified in earlier stages of HF and the response to lifestyle intervention are unknown.

We have recently demonstrated, in participants randomised to a 12-week nutritionally complete low-energy (810kCal/day) meal replacement plan (MRP), not only did 80% acheive a normoglycaemic range but there was evidence of beneficial cardiovascular reverse remodelling [12, 13]. This cohort of participants with T2D, and Stage A/B HF, provides an opportunity to explore lipidomic signals and potential underlying pathogenic mechanisms in early HF.

The aims of this *post-hoc* discovery study were to determine if; 1) following an MRP the ceramide/sphingolipid profile harmonises with a healthy profile, and if 2) the change in ceramide/sphingolipid profile is associated with reverse cardiovascular remodelling.

## Methods

**Fig. 1:**
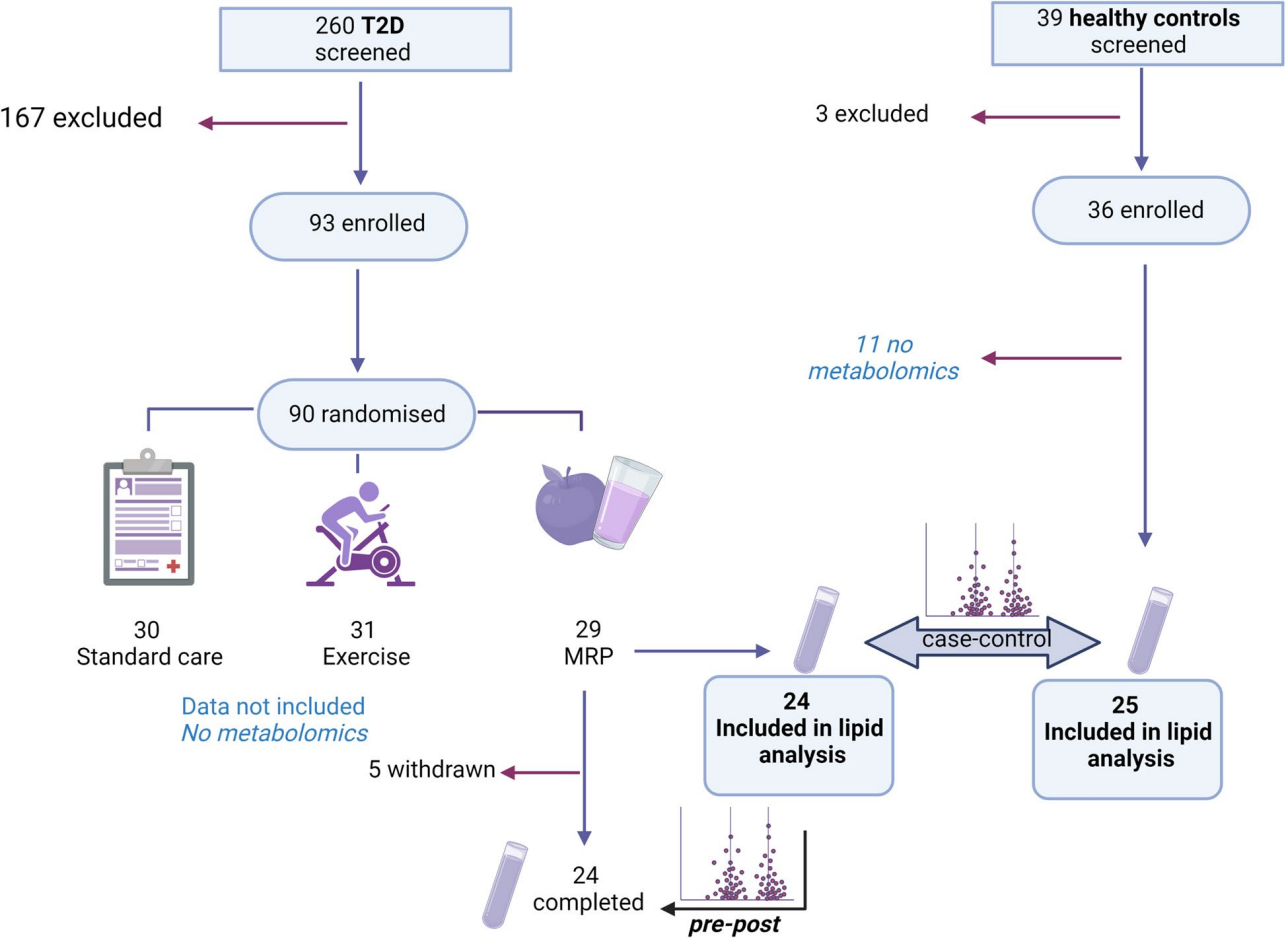
Study CONSORT diagram

This is a discovery metabolomics pilot study of the Diabetes Interventional Assessment of Slimming or Training tO Lessen Inconspicuous Cardiovascular Dysfunction (DIASTOLIC) trial. [12] DIASTOLIC was a prospective, randomised, open-label, blinded end-point trial with published protocol and main outcomes paper [12, 14]. For this *post-hoc* analysis, only those randomised to and completing the MRP were included, in addition to age-, sex- and ethnicity-matched healthy controls for baseline comparison. Participants receiving the MRP were adults (18–65yrs) with established T2D (≥ 3 months) and obesity without prevalent cardiovascular disease (CVD). The study received ethical approval by the United Kingdom National Research Ethics Service (15/WM/0222) and is registered with www.clinicaltrials.gov (NCT02590822). Consort diagram for the trial for this analysis is shown in Fig. 1.

### Demographics, anthropometrics and biochemistry

Demographics, medical history, and anthropometric measures were collected as described previously [14]. Fasting blood samples were obtained and the residual supernatant plasma stored at -80˚C prior to batch analysis. HbA1c, glucose, liver, kidney function and lipid profile were analysed according to standard operating procedures in the accredited pathology laboratory at University Hospitals Leicester NHS Trust. Insulin was quantified by multiplex assay on a Luminex platform, as previously described [14].

### Cardiovascular MRI

MRI scans were conducted on a 1.5T platform (MAGNETOM Aera; Siemens, Erlangen, Germany) as previously described [14]. Images were analysed offline blinded to treatment group. The MRI outcomes of interest (supplementary material: Cardiac MRI outcome measures) were selected to permit investigation of cardiovascular structure and function.

### Transthoracic echocardiography

Echocardiography was performed and interpreted by one of two accredited operators using an iE33 system with S5-1 transducer (Philips Medical Systems, Best, the Netherlands) to estimate Left Ventricular (LV) filling pressures (E/e').

### Metabolomics platform

Metabolon (North Carolina, USA) performed the metabolomic profiling. Samples were prepared using the automated MicroLab STAR® system from Hamilton Company. Sample analysis utilized a Waters ACQUITY ultra-performance liquid chromatography (UPLC), and a Thermo Scientific Q-Exactive high resolution/accurate mass spectrometer, interfaced with a heated electrospray ionization (HESI-II) source and Orbitrap mass analyzer operated at 35,000 mass resolution.

### Statistics

The distribution of the data were assessed for normality using histograms and Q-Q plots. Continuous data are reported as mean (± standard deviation (SD)) if normally distributed or median (interquartile range (IQR)) where not, categorical data reported as count (percentage). Demographic and standard clinical characteristics between the cases (T2D) and controls are described without testing for statistical significance given these were not specific aims being assessed. For measures of cardiovascular structure and function, between groups were tested using independent t-test and Mann–Whitney U as appropriate. The metabolomic data were restricted to lipid species to answer the specified a priori research questions. Principal component analyses (PCA) were conducted to determine if there were separation between the cases (T2D) and healthy controls (controls) at baseline, and between pre-, post-MRP in those with T2D based on the lipid profiles. The fold-change between cases and controls and between pre-, and post-MRP were calculated with adjustment for false discovery rate (FDR) of 0.05 and volcano plots generated. Candidate biomarkers were classified as lipids with FDR adjusted fold change > 1.5 (between case–control) with statistical significance *p* < 0.05. Any biomarkers with a conflict in fold-change between the two comparisons were removed. The lipids were then further restricted to sphingolipid/ceramide species because these were the lipids of interest for this pilot study. The circulating plasma levels of the candidate biomarkers post-intervention were then investigated to determine their response to the MRP. Box plots were produced to visualise the concentration (signal intensity) of candidate biomarkers pre-, post-MRP and for healthy controls. Pearson’s or Spearman’s rank correlations were used (as appropriate) to examine relationships between plasma levels of candidate biomarkers and Left Ventricular mass:volume ratio, global longitudinal strain, global circumferential strain, longitudinal peak early diastolic strain rate, circumferential peak early diastolic strain rate, myocardial perfusion reserve and diastolic function (average E/e’). Univariate linear regression was employed to explore if the change in candidate sphingolipid/ceramide species were associated with change in the same measures of cardiovascular structure and function and significant univariate associations were then adjusted for baseline value.

Data were analysed using IBM SPSS Statistics for Windows, Version 26.0 and RStudio version 1.4. SIMCA version 14 (MSK Umetrics, Sweden) was used to perform PCA plots.

## Results

### Case–control analysis

Twenty-nine asymptomatic T2D participants (cases) were randomised to the MRP group with 24 completing the study with metabolomics data. Twenty-five matched controls (age, sex and ethnicity) were selected for metabolomics analysis (Fig. 1). The baseline characteristics by group are provided in Table 1. At baseline, as expected, cases had higher body weight, blood pressure, HbA1c and insulin resistance with over half having hypertension and high cholesterol compared to none in the healthy group. Diabetes medication included one person with diet and lifestyle only (4%), 18 (75%) on monotherapy, three (13%) on dual therapy and two (8%) on triple therapy. No patients were taking insulin and only one and two were on Glucagon Like Peptide-1-Receptor agonists or Sodium-Glucose co-transporter 2 inhibitors, respectively. None of the healthy controls were taking any medications.

**Table 1:** Characteristics table (cases and control)

**Variable**	**Cases (T2D) *N* = 24**	**Controls (healthy) *N* = 25**	
Clinical characteristics			
Age (years)	51.1 ± 5.7	48.3 ± 6.6	
Sex (M (%))	15 (63)	15 (60)	
Ethnicity (WE (%))	15 (63)	17 (68)	
Weight (kg)	106.7 ± 16.2	69.9 ± 11.4	
BMI (kg/m2)	37.4 ± 5.9	24.3 ± 2.5	
Systolic BP (mmHg)	145.9 ± 15.9	118.4 ± 10.7	
Diastolic BP (mmHg)	91.1 ± 7.4	76.2 ± 6.0	
Heart rate (bpm)	73.1 ± 8.6	61.8 ± 9.3	
HbA1c (%)	7.2 ± 1.1	5.4 ± 0.2	
HbA1c (mmol/mol)	54.8 ± 11.9	35.6 ± 2.6	
HOMAR-IR	12.2 ± 8.2	2.0 ± 1.9	
Medical history			
Duration diabetes (m)	58.3 ± 39.8	-	
Smoking history (yes)	10 (41)	7 (28)	
Hypertension (n (%))	15 (63)	0 (0)	
Hypercholesterolemia (n (%))	16 (67)	0 (0)	
Cardiovascular Imaging			*P* value
LV Massi (g/m^2^)	58.2 ± 9.8	57.8 ± 13.1	0.902
LV EDVi (mL/m^2^)	69.9 ± 11.3	82.9 ± 18.0	**0.004**
LV mass:volume (g/mL)	0.84 ± 0.13	0.70 ± 0.08	**< 0.001**
LV EF (%)	70.0 ± 7.4	64.7 ± 4.9	0.005
LV GLS (%)	16.6 ± 2.8	17.6 ± 1.5	0.108
LV GCS (%)	21.0 ± 2.3	19.6 ± 2.0	**0.029**
LV LongPEDSR (s-1)	0.79 ± 0.15	0.89 ± 0.16	**0.021**
LV CircPEDSR (s-1)	1.00 ± 0.20	1.10 ± 0.16	0.060
LAVImax (mL/ m2)*	30.4 (10.4)	50.6 (16.8)	**< 0.001**
LA EF (%)	55.0 ± 7.7	59.1 ± 7.8	0.075
Mean Ao Distens (mmHg^−1^ × 10^–3^)	3.7 ± 1.9	6.9 ± 2.0	**< 0.001**
MPR	3.0 ± 1.0	4.2 ± 1.0	**< 0.001**
Average E/e'*	9.4 (4.5)	5.9 (2.6)	**< 0.001**

Data are reported as mean ± standard deviation, count (percent) or median (interquartile range). Bold font highlights statistically significant difference with significance level of 95%

*Abbreviations*: *BMI* body mass index, *LV EF* Left ventricular ejection fraction, *LV Massi* Left Ventricular Mass Indexed for body surface area, *mass:volume *mass to volume ratio, *LV EDVi* Left Ventricular End Diastolic Volume indexed for height, *GLS* global longitudinal strain, *LongPEDSR* Longitudinal Peak Early Diastolic Strain Rate, *CircPEDSR* Circumferential Peak Early Diastolic Strain Rate, *GCS* global circumferential strain, *LAVImax* Left atrial maximum volume indexed for body surface area, *Mean Ao Distens* Mean aortic distensibility, *MPR* Myocardial perfusion reserve

There was evidence of cardiovascular remodelling in those with T2D who had significantly lower left ventricular volume, higher mass:volume ratio with higher ejection fraction and global strain but worse diastolic function (LV filling pressures (E/é) and peak early diastolic strain rate). In addition, there was decreased aortic distensibility and lower myocardial perfusion reserve in T2D compared to the controls.

### Lipids

**Fig. 2:**
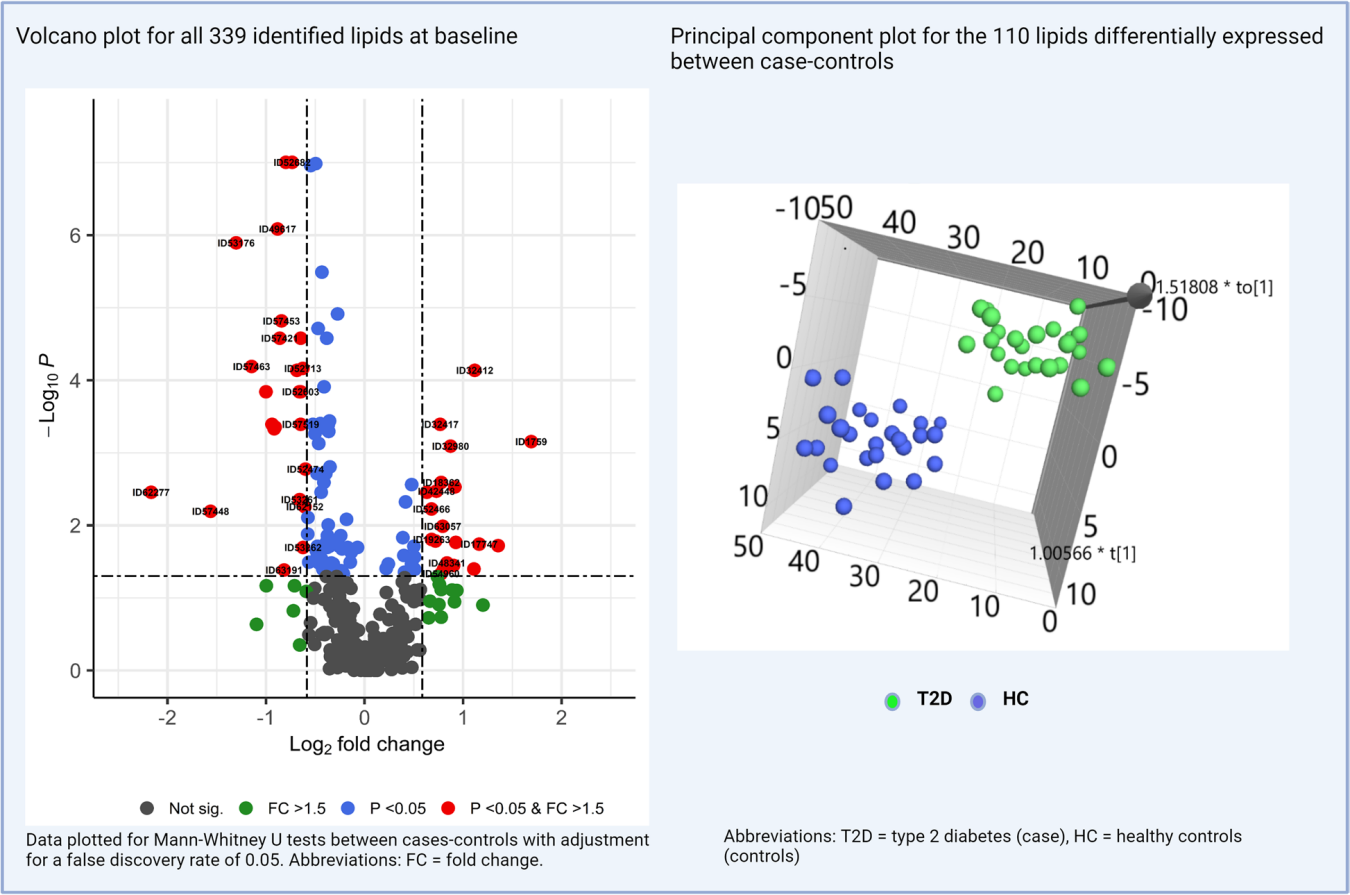
Volcano plot for all 339 identified lipids at baseline. Principal component plot for the 110 lipids differentially expressed between cases-controls

The metabolon platform returned 339 lipids species, of which 110 were significantly different between cases and controls (Fig. 2a); 78 were significantly downregulated and 32 significantly upregulated in cases versus controls. There was good separation in discriminating between the groups based on these lipid species (Fig. 2b) of which a total of forty-four had a fold-changes > 1.5 meeting statistical significance adjusted for FDR of 0.05 (Fig. 2a: red-points only).

### Pre-post MRP

Twenty-four cases completed the MRP intervention and had plasma for metabolomic analysis. There were significant cardio-metabolic improvements including weight loss (13.6kg), reduced blood pressure (13mmHg systolic), reduced arterial stiffness, reduced concentric remodelling, decreased insulin resistance and fasting glucose (-1.9mmol), with 20 (83%) participants in this group achieving normoglycaemia by 12 weeks (Supplemental material Table S1), as previously reported [12].

**Fig. 3:**
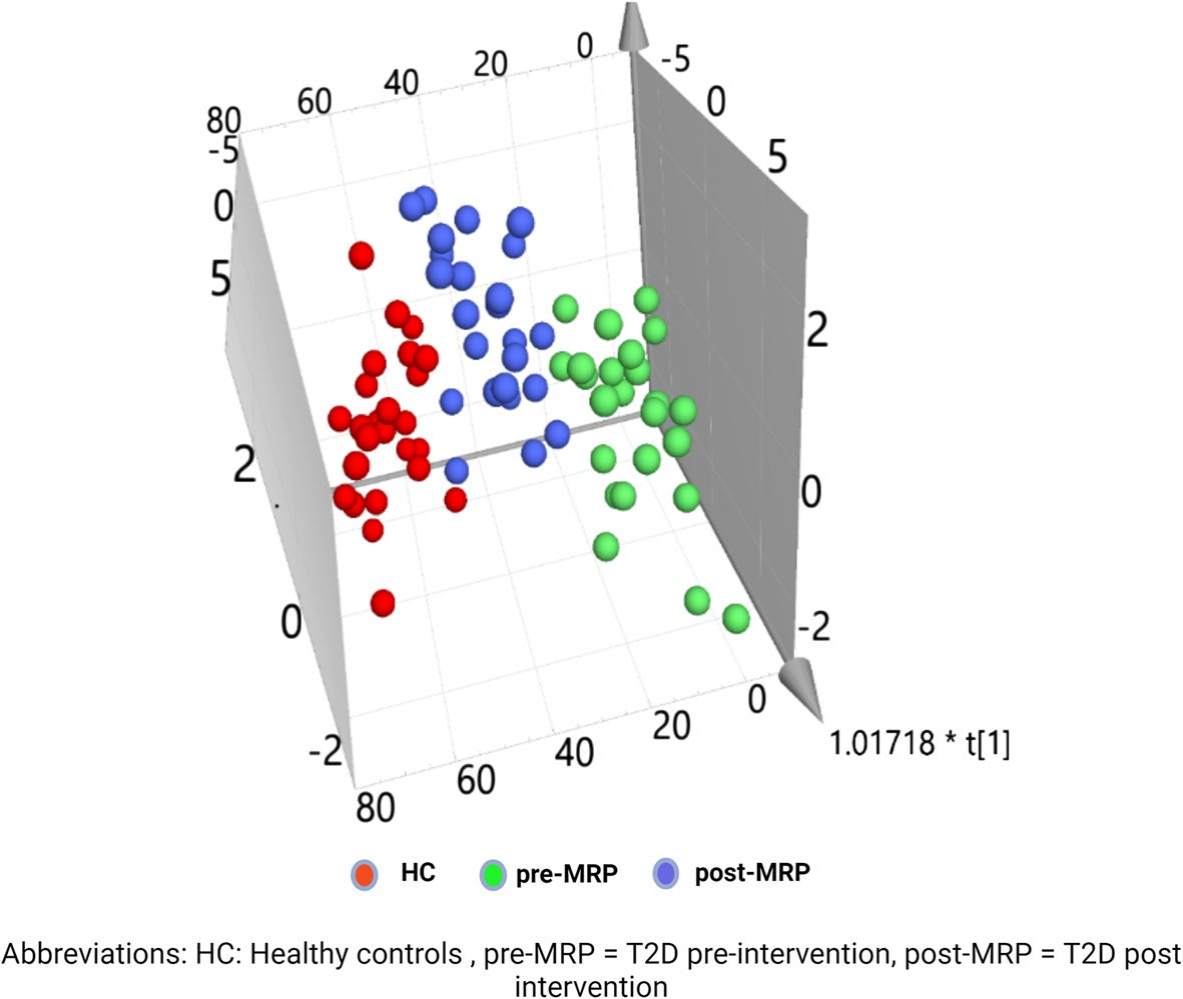
Principal component plot for all 23 identified lipids for case (pre-MRP and post-MRP) and healthy controls

**Fig. 4:**
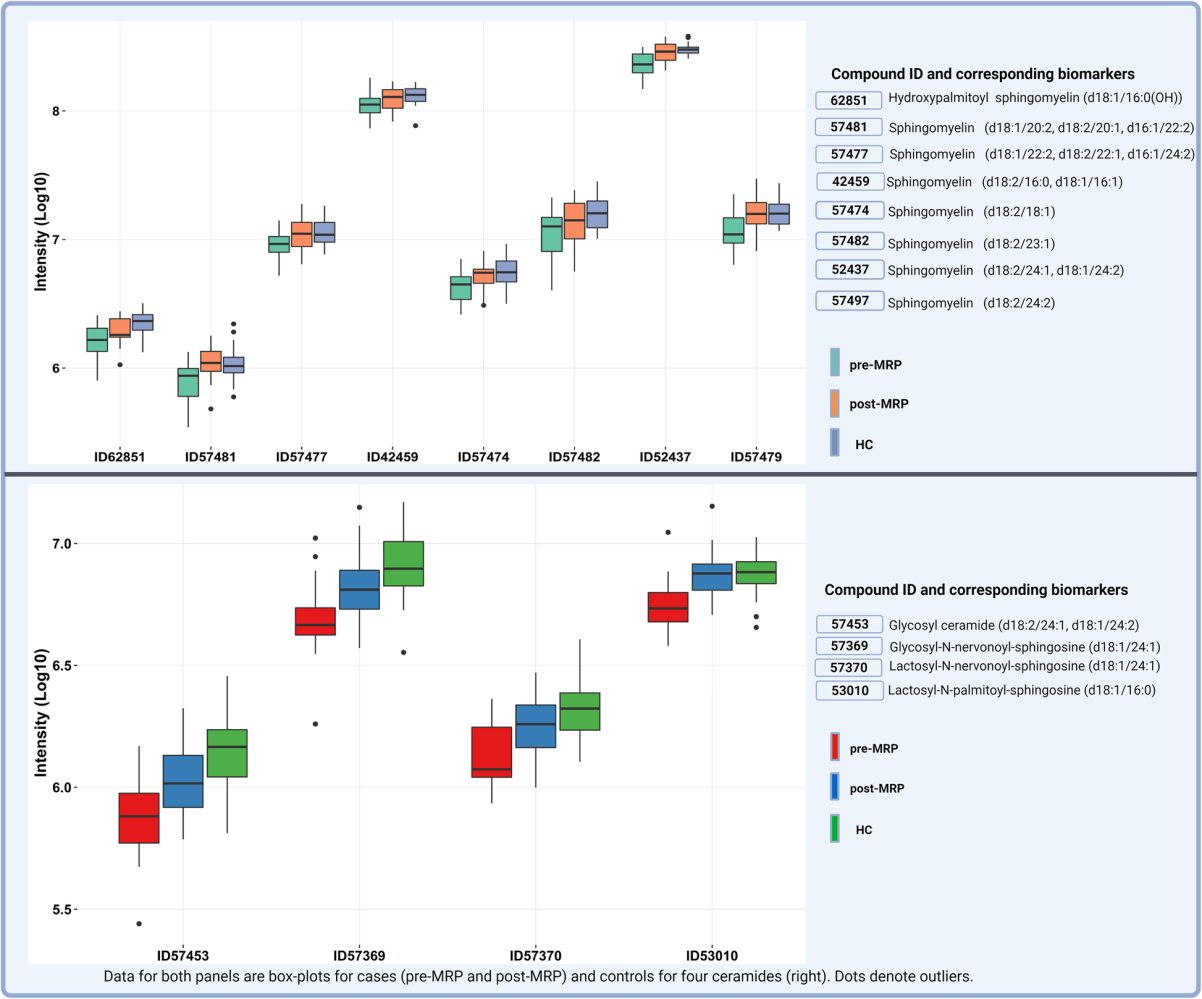
Box plots for plasma levels of candidate sphingolipids and ceramides for Type 2 Diabetes (pre-, post-MRP) and healthy controls

Of the 110 candidate biomarkers identified in the case–control comparison, 25 were significantly different following the 12-week MRP. Following removal of conflicts in fold-change between the comparisons a final 23 candidate biomarkers were identified (Table 2). Restricting analyses to these 23 candidate biomarkers there is a good separation between pre-, post-MRP (Fig. 3; Green and blue dots only) and evidence of a shift towards the healthy volunteers following the MRP (Fig. 3; Green, blue and red dots). We then further restricted the analysis to the sphingolipids and ceramides species (Table 2), given the focus of these analysis. All 12 species contain one long chain (> 16 Carbon atoms) unsaturated (≥ 1 double bond) fatty acid. The aforementioned shift towards healthy levels is observed in all the 12 species as demonstrated in box-plots in Fig. 4a and b. All 12 of these candidate biomarkers were significantly lower at baseline and increased to near ‘normal’ levels following the 12-week MRP.

**Table 2:** List of 23 lipid candidate biomarkers with significant fold-change > 1.5 following the 12-week MRP

*No*	*BIOCHEMICAL*	*COMP ID*	*Sub-family*	*Fold Change*	*Direction Δ from BL*	*Adjusted P value (FDR* = *0.05)*
*1*	nisinate (24:6n3)	57810	Long Chain Polyunsaturated Fatty Acid (n3 and n6)	-2.08	↓	0.005
*2*	1-(1-enyl-palmitoyl)-2-linoleoyl-GPC (P-16:0/18:2)	52682	Plasmalogen	0.81	↑	0.002
*3*	1-(1-enyl-palmitoyl)-2-oleoyl-GPC (P-16:0/18:1)	52478	Plasmalogen	0.71	↑	< 0.001
*4*	1-(1-enyl-palmitoyl)-2-palmitoleoyl-GPC (P-16:0/16:1)	52713	Plasmalogen	0.77	↑	< 0.001
*5*	1-(1-enyl-palmitoyl)-2-palmitoyl-GPC (P-16:0/16:0)	52716	Plasmalogen	0.80	↑	0.003
*6*	1-(1-enyl-palmitoyl)-GPC (P-16:0)	52474	Lysoplasmalogen	0.64	↑	0.004
*7*	1-palmitoyl-2-oleoyl-GPE (16:0/18:1)	19263	Phosphatidylethanolamine (PE)	-1.55	↓	0.003
*8*	1-stearoyl-2-oleoyl-GPE (18:0/18:1)	42448	Phosphatidylethanolamine (PE)	-1.88	↓	0.001
*9*	butyrylcarnitine (C4)	32412	Fatty Acid Metabolism	-1.39	↓	0.005
*10*	dihomo-linoleoylcarnitine (C20:2)	57520	Fatty Acid Metabolism (Acyl Carnitine, Polyunsaturated)	0.72	↑	0.003
*11*	eicosenoylcarnitine (C20:1)	57519	Fatty Acid Metabolism (Acyl Carnitine, Monounsaturated)	0.69	↑	0.002
*12*	hydroxypalmitoyl sphingomyelin (d18:1/16:0(OH))	62851	Sphingomyelins	0.85	↑	0.004
*13*	sphingomyelin (d18:1/20:2, d18:2/20:1, d16:1/22:2)	57481	Sphingomyelins	0.70	↑	0.001
*14*	sphingomyelin (d18:1/22:2, d18:2/22:1, d16:1/24:2)	57477	Sphingomyelins	0.80	↑	0.002
*15*	sphingomyelin (d18:2/16:0, d18:1/16:1)	42459	Sphingomyelins	0.89	↑	0.004
*16*	sphingomyelin (d18:2/18:1)	57474	Sphingomyelins	0.83	↑	0.004
*17*	sphingomyelin (d18:2/23:1)	57482	Sphingomyelins	0.85	↑	0.007
*18*	sphingomyelin (d18:2/24:1, d18:1/24:2)	52437	Sphingomyelins	0.80	↑	< 0.001
*19*	sphingomyelin (d18:2/24:2)	57479	Sphingomyelins	0.73	↑	< 0.001
*20*	glycosyl ceramide (d18:2/24:1, d18:1/24:2)	57453	Hexosylceramides (HCER)	0.71	↑	0.001
*21*	glycosyl-N-nervonoyl-sphingosine (d18:1/24:1)	57369	Hexosylceramides (HCER)	0.72	↑	< 0.001
*22*	lactosyl-N-nervonoyl-sphingosine (d18:1/24:1)	57370	Lactosylceramides (LCER)	0.76	↑	0.001
*23*	lactosyl-N-palmitoyl-sphingosine (d18:1/16:0)	53010	Lactosylceramides (LCER)	0.76	↑	< 0.001

Lipids with statistically significant fold-change pre- to post-MRP (calculated as baseline/week 12)

### Cardiac remodelling and candidate biomarkers

In the baseline correlation analysis, for the pooled subjects (n = 49), three candidate sphingolipids and the four ceramide species were inversely correlated with LV mass: volume ratio (Table 3). Four sphingolipids and one ceramide were positively correlated to longitudinal PEDSR. In addition to these species a further three sphingolipids (total of seven) and the same ceramide species were also positively correlated to circumferential PEDSR. Finally, the ceramide, lactosyl-N-palmitoyl-sphingosine, was positively correlated with MPR and negatively correlated with LV filling pressure (E/e’).

**Table 3:** Baseline correlations between sphingolipids and ceramides and key measures of cardiac structure and function

Biomarker	LV m:v	GLS	GCS	LongPEDSR	CircPEDSR	MPR	E/e’
***Sphingomyelin species***							
Hydroxypalmitoyl sphingomyelin (d18:1/16:0(OH))	**-0.348**^**a**^	0.117	0.042	0.226	**0.463**^**b**^	0.105	-0.207
Sphingomyelin (d18:2/16:0, d18:1/16:1)	**-0.320**^**a**^	0.123	-0.032	0.279	**0.325**^**a**^	0.033	0.330
Sphingomyelin (d18:2/24:1, d18:1/24:2)	**-0.384**^**b**^	0.211	-0.185	**0.336**^**a**^	**0.408**^**b**^	0.263	-0.198
Sphingomyelin (d18:1/22:2, d18:2/22:1, d16:1/24:2)	-0.177	0.201	0.032	**0.368**^**b**^	**0.423**^**b**^	0.037	0.084
Sphingomyelin (d18:1/20:2, d18:2/20:1, d16:1/22:2)	-0.178	0.090	-0.048	0.249	**0.361**^**a**^	-0.047	0.063
Sphingomyelin (d18:2/18:1)	-0.188	0.057	-0.088	0.233	0.236	0.040	0.001
Sphingomyelin (d18:2/23:1)	-0.138	0.207	-0.032	**0.384**^**b**^	**0.378**^**b**^	0.122	0.089
Sphingomyelin (d18:2/24:2)	-0.264	0.216	-0.003	**0.374**^**b**^	**0.419**^**b**^	-0.037	0.020
***Ceramide species***							
Glycosyl ceramide (d18:2/24:1, d18:1/24:2)	**-0.388**^**b**^	0.139	-0.137	**0.344**^**b**^	**0.340**^**a**^	0.212	-0.165
Glycosyl-N-nervonoyl-sphingosine (d18:1/24:1)^+^	**-0.395**^**b**^	0.063	-0.167	0.260	0.124	0.186	-0.058
Lactosyl-N-nervonoyl-sphingosine (d18:1/24:1)	**-0.473**^**b**^	0.079	-0.244	0.129	0.102	0.268	-0.266
Lactosyl-N-palmitoyl-sphingosine (d18:1/16:0)	**-0.458**^**b**^	-0.074	-0.226	-0.002	0.064	**0.312**^**a**^	**-0.363**^**a**^

Data reported are Spearmans Rank correlation co-efficient or ^+^Pearsons correlation coefficient

*Abbreviations*: *LV* left ventricular, *m:v* mass to volume ratio, *GLS* global longitudinal strain, *GCS* global circumferential strain, *LongPDESR* longitudinal peak early diastolic function, *Circ* circumferental, *MPR* myocardial perfusion reserve, *E/e’* diastolic function

^a^Correlation is significant at the 0.05 level

^b^Correlation is significant at the 0.01 level

The results of the univariate analysis are displayed in Table 4. There was no association between change in LV mass:volume ratio, change in circumferential PEDSR, change in MPR nor change in E/e’ and change in sphingolipid/ceramide species that were shown to be significantly correlated at baseline. However, there was an inverse relationship between change in two sphingolipids and change in longitudinal PEDSR (Table 4) which did not remain significant after adjustment for baseline values (β = -0.25 (95%CI: -0.50, -0.00), *p* = 0.05 and β = -0.16 (95%CI: -0.32, 0.01), *p* = 0.06 for Sphingomyelin (d18:2/16:0, d18:1/16:1) and Sphingomyelin (d18:2/24:2), respectively).

**Table 4:** Relationship between change in LV mass/LVmass:volume ratio and strain rates and change in sphingolipids and ceramides at 12-weeks post MRP

	**Β coefficient (95%CI)**	***p* value**	**Β coefficient (95%CI)**	***p* value**	**Β coefficient (95%CI)**	***p* value**	**Β coefficient (95%CI)**	***p* value**	**Β coefficient (95%CI)**	***p* value**
***Δ* Candidate Biomarker**	***Δ LV mass***		***Δ LongPEDSR***		***Δ CircPEDSR***		***Δ MPR***		***Δ E/e’***	
***Sphingomyelin species***										
Hydroxypalmitoyl sphingomyelin (d18:1/16:0(OH))	9.65 (-17.17, 36.46)	0.481			-0.04 (-0.39, 0.31)	0.820				
Sphingomyelin (d18:2/16:0, d18:1/16:1)	4.43(-30.90, 39.74)	0.806	-0.31 (-0.56, -0.07)	0.013*	-0.26 (-0.70, 0.19)	0.260				
Sphingomyelin (d18:2/24:1, d18:1/24:2)	7.51(-28.00, 43.02)	0.678	-0.23 (-0.49, 0.04)	0.098	-0.10 (-0.56, 0.36)	0.672				
Sphingomyelin (d18:1/22:2, d18:2/22:1, d16:1/24:2)	-7.25 (-29.94, 15.45)	0.531	-0.16 (-0.33, 0.00)	0.056	-0.20 (-0.48, 0.08)	0.164				
Sphingomyelin (d18:1/20:2, d18:2/20:1, d16:1/22:2)	-7.01 (-24.08, 10.07)	0.421			-0.11 (-0.33, 0.11)	0.326				
Sphingomyelin (d18:2/18:1)	-8.04(-30.59, 14.52)	0.485								
Sphingomyelin (d18:2/23:1)	-2.55(-27.39, 22.29)	0.841			0.70 (-0.25, 0.39)	0.669				
Sphingomyelin (d18:2/24:2)	-4.33(-26.50, 17.85)	0.702	-0.17 (-0.33, -0.01)	0.037*	-0.16 (-0.44, 0.12)	0.275				
***Ceramide species***										
Glycosyl Ceramide (d18:2/24:1, d18:1/24:2)	-3.71 (-21.62, 14.19)	0.685	-0.12 (-0.25, 0.01)	0.081	0.15 (-0.07, 0.37)	0.189				
Glycosyl-N-nervonoyl-sphingosine (d18:1/24:1)	2.30 (-19.79, 24.39)	0.838								
Lactosyl-N-nervonoyl-sphingosine (d18:1/24:1)	4.05(-19.96, 28.06)	0.741								
Lactosyl-N-palmitoyl-sphingosine (d18:1/16:0)	2.42(-27.84, 32.68)	0.876					0.06 (-0.01, 0.13)	0.068	0.01 (-0.01, 0.03)	0.202

*Abbreviations*: *CI* Confidence Interval, *Δ change, LV* Left Ventricular, *M:V* mass to volume ratio, *Long* longitudinal, *PEDSR* Peak Early Diastolic Strain Rate, *Circ* Circumferential, *MPR* Myocardial Perfusion Reserve, *E/e’* diastolic function

^*^P ≥ 0.05 following adjustment for baseline values

## Discussion

In this pilot discovery study 12 lipid species were identified, eight sphingolipids and four ceramides, in the asymptomatic T2D cohort, that were down regulated at baseline and following a 12-week MRP increased towards healthy control levels. Of these 12 candidate lipids; there were negative correlations between seven and LV mass/volume ratio and one with LV E/e’ and positive correlations between five and longitudinal PEDSR, eight and circumferential PESDR and one with MPR. This data indicates that high levels of these long-chained unsaturated sphingolipid and ceramide species are associated with less concentric remodelling (lower LV mass:volume ratio), better myocardial microvascular function (higher MPR) and better diastolic function (higher PEDSR and lower E/e’). However, following the MRP the change in only two circulating plasma sphingolipids were associated with a change in diastolic strain rate, specifically a reduction in diastolic strain rate (lower longitudinal PESDR), but this did not remain significant when adjusting for baseline values. Collectively these data cast doubt on the putative causative role of these lipid molecules in the development of HFpEF.

There are many ceramide and sphingomyelin species, determined by the specifics of the fatty acids they carry and can be grouped into long and short chain species, which lends itself to the myriad of functions these lipids have. The sphingolipids, shown to be significantly reduced in our participants with T2D, are involved in numerous cellular processes that could be involved in the development of HFpEF in T2D including; cell cycle arrest, apoptosis, senescence and other stress responses [15]. This is in addition to a number of important biophysiological processes including; oxidative stress and inflammation [16], endothelial dysfunction [17], lipotoxicity [18], and insulin resistance [19] which may also play a role in the pathogenesis of HFpEF in T2D. Indeed, there has been an emergence of evidence linking these bioactive lipids to the development of chronic conditions such as T2D or HF [20, 21]. The majority of the evidence in humans is derived from large prospective studies with the associated risk thought to be determined by the composition of the fatty acid moiety. That is the length (number of carbons) and number of double bonds present in the fatty acid chains of the sphingolipid/ceramide species, specifically, longer chained saturated fatty acids are reportedly associated with lower risk [22]. Notably, each species identified in our study contained at least one long-chain fatty acid that was polyunsaturated.

### Case–control

Our data show lower levels of circulating long-chain polyunsaturated ceramide and sphingolipid species in adults with asymptomatic T2D (Stage A/B HF) compared to healthy controls. The Cardiovascular Health Study (CHS) [21] reported that longer-chain sphingolipids (Cer-20 and -22, SM-20 and -22) are associated with a lower risk of HF even after adjustment for traditional risk factors and shorter chain species (Cer-16) irrespective of HF type. Previously, 24 HFpEF patients were compared with 38 aged matched controls without a history of heart failure from the Alberta HEART study [11]. In line with our findings they found 14 sphingomyelin species, of which 12 were longer chain and/or polyunsaturated (C16.1 to C26.0/1), to be down regulated in those with established HFpEF compared to controls. Cheng et al., have also reported significantly lower plasma levels of sphingomyelin (C20:2) in 73 Stage C HF *vs*. 51 controls [23]. This supports the baseline correlations we observed between higher circulating levels of the long-chain sphingolipid/ceramide species and less concentric remodelling (lower LV mass:volume ratio), better myocardial microvascular function (higher MPR) and better diastolic function (higher PEDSR and lower E/e’).

In a larger cohort of patients with HFpEF (*n* = 282) compared to non-HF controls (*n* = 191) from the CATHGEN biorepository [24], evidence of impaired or dysregulated fatty acid oxidation in HFpEF was reported. In their targeted mass spectrometry study, they quantified 63 metabolites (45 acylcarnitines and 15 amino acids) and reported long-chain acylcarnitine’s to be significantly higher in HFrEF than HFpEF, with levels increasing linearly with declining left ventricular ejection fraction [25]. The key functions of carnitine and its derivatives are to; 1) shuttle long-chain fatty acids across the mitochondrial membrane for energy generation via β-oxidation and, 2) to act as a scavenger by binding and eliminating acyl residues generated from amino-acid metabolism [26]. These results are contrary to our own for the three identified carnitine species. The conflicting results could be attributed to the difference in clinical characteristics between the cohorts studied with participants from the CATHGEN biorepository being older, greater white European representation and with overt HFpEF. Our data are suggestive of dysregulated fatty acid oxidation in asymptomatic T2D who fit the classification for Stage A/B HF compared to HC.

### Cardiac remodelling and candidate biomarkers

We found moderate inverse correlations between four ceramide and three sphingomyelin species and CMR derived LV mass/volume ratio which may indicate involvement of these lipid species in cardiovascular remodelling in T2D. LV mass/volume ratio is a key measure of cardiac concentric remodelling, an important structural abnormality in the early stages of HF [2], and an adverse prognostic factor in HFpEF [27, 28]. There was evidence of concentric remodelling within our cohort of asymptomatic T2D (higher mass/volume ratio) [12].

Data from both animal and human models link sphingolipid accumulation in cardiomyocytes with cardiac hypertrophy [29]. However, plasma sphingolipids are reflective of systemic sphingolipid levels and not localised levels. Thus, from the data presented here it cannot be deduced what level or indeed the composition of sphingolipids that lie within the myocardium of this cohort. The high level of circulating long-chain polyunsaturated sphingolipids may be indicative of localised shorter chain saturated sphingolipids within the myocardium. This observed inverse relationship requires confirmation in larger cohorts in conjunction with a more comprehensive multivariable analysis.

### Pre-, post-intervention

To our knowledge, this is the first study to demonstrate an increase in circulating sphingolipid and ceramide species following a low-energy diet, as part of a randomised controlled trial, in asymptomatic T2D fitting the classification of Stage A/B HF. Furthermore, the increase in levels appear to have moved towards HC levels as demonstrated by the reduced separation observed in the 3D-visulisation between HC and MRP post-intervention vs. baseline (PCA). Strikingly, the observed increase was significant across all the identified sphingolipid and ceramide species. However, we found no significant association between changes in circulating levels of these species and measures of cardiac structure and function therefore casting doubt on the putative causative role of these lipid molecules in the development of HFpEF. Perhaps it is the flux of the sphingolipid/ceramide species i.e.; the ratio of short:long chain species, that is important in the pathogenesis of HFpEF, which cannot be answered by these data, but warrants further exploration in larger longitudinal studies with targeted lipidomic analysis.

### Strengths and limitations

The major strength and novelty of this study is that we utilised samples from participants in the DIASTOLIC randomised controlled trial, with well-balanced group allocation in addition to matched healthy controls at baseline. The metabolomic data analyses was conducted blinded to group allocation. To our knowledge this is the first metabolomic analysis of a lifestyle intervention in people with asymptomatic T2D with Stage A/B HF classification that includes detailed cardiovascular structural and functional phenotyping with the gold standard technique of CMR. However, we acknowledge the main limitation relates to this study being a post-hoc analysis with limited sample size and not including all groups of the RCT due to limited funding. Metabolomic studies are a “snap-shot” in time and cannot deduce the cause for the observed levels i.e.; a metabolite could be lower because of decreased production, higher degradation and /or uptake, or both. Furthermore, circulating levels may not reflect myocardial concentrations, which are incredibly difficult to obtain from asymptomatic individuals. The metabolites identified in this pilot study require verification in a larger, prospective, validation study.

## Conclusion

Working aged adults with asymptomatic T2D and Stage A/B HF classification have impaired or dysregulated fatty-acid metabolism represented by reduced levels of long-chain-polyunsaturated sphingomyelin/ceramide species. However, no association between the changes in circulating levels of these species and reverse cardiac remodelling were observed. Whilst this may cast doubt on the putative causative role of these lipid species in the development of HFpEF in T2D, the reverse remodelling observed in this cohort was mild and our sample size was small, therefore we may not be sufficiently powered to detect such a relationship. The findings from this pilot work require confirmation in a larger, prospective external validation cohort with a targeted lipidomic approach.

### Supplementary Information

**Additional file 1:** **Table S1.** T2D baseline characteristics and change at 12 weeks post intervention.

## Data Availability

The datasets used and/or analysed during the current study are available from the corresponding author on reasonable request.
