# Supplementary material for: Circulating sphingolipids and relationship to cardiac remodelling before and following a low-energy diet in asymptomatic Type 2 Diabetes
Source: BMC Cardiovasc Disord. 2024 Jan 3;24:25. doi: 10.1186/s12872-023-03623-y (PMC10765891; doi:10.1186/s12872-023-03623-y)
Supplement: Supplementary file 1 — Additional file 1: Table S1. T2D baseline characteristics and change at 12 weeks post intervention. [file 12872_2023_3623_MOESM1_ESM.docx]

# Supplemental material

### Cardiac MRI outcome measures

The following CMR outcomes of interest were analysed: LV End Diastolic Volume (LV EDV (ml)), LV mass (LVM, g), LV mass to volume ratio (LV mass/volume (g/mL), LV Global Longitudinal Strain ( LV GLS (%)), LV Global Circumferential Strain (LV GCS (%)), LV Longitudinal Peak Early Diastolic Strain rate (LV LongPEDSR (s-1)), LV Circumferential Peak Early Diastolic Strain rate (LV CircPEDSR (s-1)), Left Atrial maximal volume (LAmax V (mL/m2)), LA Ejection Fraction (LA EF (%)), LV Ejection Fraction (LV EF (%)), mean aortic distensibility (mmHg-1 x 10-3), myocardial perfusion reserve (MPR).^3^

### **Table S1**: T2D baseline characteristics and change at 12 weeks post intervention

| **Variable** | **Baseline**  **N=24** | **Change from baseline**  **N=24** | ***p* value** |
| --- | --- | --- | --- |
| **Clinical characteristics** | | | |
| Weight (kg) | 106.7 ± 16.2 | -13.7 ± 4.8 | **<0.001** |
| BMI (kg/m^2^) | 37.4 ± 5.9 | -4.7 ± 1.7 | **<0.001** |
| SysBP (mmHg) | 145.9 ± 15.9 | -13.0 ± 20.4 | **0.005** |
| DiaBP (mmHg) | 91.1 ± 7.4 | -4.7 ± 11.5 | 0.058 |
| Heart rate (bpm) | 73.1 ± 8.6 | -5.3 ± 7.7 | **0.003** |
| HbA1c (%) | 7.2 ± 1.1 | -0.96 ± 0.95 | **<0.001** |
| HOMAR-IR | 12.2 ± 8.2 | -7. 5 ± 8.4 | **<0.001** |
| **Cardiovascular structure and function** | | | |
| LV EF (%) | 70.0 ± 7.4 | 4.5 ± 5.5 | 0.001 |
| LV Mass (g) | 131.2 ± 26.9 | -5.6 ± 13.8 | 0.066 |
| LV mass:volume (g/mL) | 0.84 ± 0.13 | -0.03 ± 0.06 | **0.017** |
| LV EDV (mL/) | 159.4 ± 31.2 | -0.2 ± 13.3 | 0.958 |
| LV GLS (+%) | 16.6 ± 2.8 | -0.6 ± 2.6 | 0.271 |
| LV GCS (+%) | 21.0 ± 2.3 | -1.3 ± 2.4 | **0.016** |
| LV LongPEDSR (s-1) | 0.79 ± 0.15 | -0.06 ± 0.11 | **0.012** |
| LV CircPEDSR (s-1) | 1.00 ± 0.20 | -0.05 ± 0.18 | 0.199 |
| LAVmax (mL/)* | 81.7 (36.3) | -5.7 (24.0) | 0.903 |
| LA EF (%) | 55.0 ± 7.7 | 0.33 ± 7.02 | 0.823 |
| Ao Distensibility  (mmHg^-1^x10^-3^) | 3.7 ± 1.9 | 0.90 ± 1.2 | **0.001** |
| MPR | 3.0 ± 1.0 | 0.2 ± 1.2 | 0.550 |
| E/e'* | 9.4 (4.5) | -0.6 (3.3) | 0.204 |

Abbreviations: BMI = body mass index, sys = systolic, dia = diastolic, SGLT2i = sodium glucose co-transporter 2 inhibitor, GLP1-RA = glucagon-like peptide 1 receptor agonist, DPP-IV = dipeptidyl peptidase-4, LV EF = Left ventricular ejection fraction, LV Mass = Left Ventricular Mass, mass:volume = mass to volume ratio , LV EDV = Left Ventricular End Diastolic Volume, GLS = global longitudinal strain, LongPEDSR = Longitudinal Peak Early Diastolic Strain rate, CircPEDSR = Circumferential Peak Early Diastolic Strain Rate, GCS = global circumferential strain, LAVmax = Left atrial maximum volume, Ao=Aortic;, MPR= Myocardial perfusion reserve. Data are reported as mean ± standard deviation, count (percent) or median (interquartile range). Bold font highlights statistically significant difference with significance level p=0.05.
